# Increased rate of hair keratin gene loss in the cetacean lineage

**DOI:** 10.1186/1471-2164-15-869

**Published:** 2014-10-06

**Authors:** Mariana F Nery, José Ignacio Arroyo, Juan C Opazo

**Affiliations:** Instituto de Ciencias Ambientales y Evolutivas, Facultad de Ciencias, Universidad Austral de Chile, Valdivia, Chile; Departamento de Biologia, FFCLRP, Universidade de São Paulo, Ribeirão Preto, Brazil; Programa de Doctorado en Ciencias mención Ecología y Evolución, Facultad de Ciencias, Universidad Austral de Chile, Valdivia, Chile

**Keywords:** Alpha keratin, Adaptation, Gene family evolution, Cetaceans, Mammals, Pseudogenes

## Abstract

**Background:**

Hair represents an evolutionary innovation that appeared early on mammalian evolutionary history, and presumably contributed significantly to the rapid radiation of the group. An interesting event in hair evolution has been its secondary loss in some mammalian groups, such as cetaceans, whose hairless phenotype appears to be an adaptive response to better meet the environmental conditions. To determine whether different repertoire of keratin genes among mammals can potentially explain the phenotypic hair features of different lineages, we characterized the type I and II clusters of alpha keratins from eight mammalian species, including the hairless dolphin and minke whale representing the order Cetacea.

**Results:**

We combined the available genomic information with phylogenetic analysis to conduct a comprehensive analysis of the evolutionary patterns of keratin gene clusters. We found that both type I and II gene clusters are fairly conserved among the terrestrial mammals included in this study, with lineage specific gene duplication and gene loss. Nevertheless, there is also evidence for an increased rate of pseudogenization in the cetacean lineage when compared to their terrestrial relatives, especially among the hair type keratins.

**Conclusions:**

Here we present a comprehensive characterization of alpha-keratin genes among mammals and elucidate the mechanisms involved in the evolution of this gene family. We identified lineage-specific gene duplications and gene loss among the Laurasiatherian and Euarchontoglires species included in the study. Interestingly, cetaceans present an increased loss of hair-type keratin genes when compared to other terrestrial mammals. As suggested by the ‘less-is-more’ hypothesis, we do not rule out the possibility that the gene loss of hair-type keratin genes in these species might be associated to the hairless phenotype and could have been adaptive in response to new selective pressures imposed by the colonization of a new habitat. Our study provides support for the idea that pseudogenes are not simply ‘genomic fossils’ but instead have adaptive roles during the evolutionary process.

**Electronic supplementary material:**

The online version of this article (doi:10.1186/1471-2164-15-869) contains supplementary material, which is available to authorized users.

## Background

**Figure 1:**
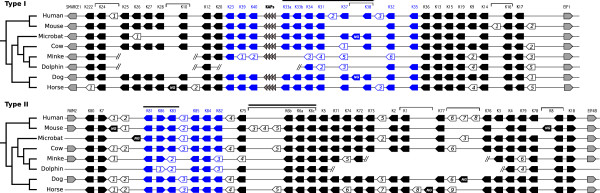
**Genomic organization of type I and type II keratins.** Direction of the arrows indicate the direction of keratin genes in the genome. Protein names follow [8] for human keratins, and are numbered according to their genomic position. Connecting lines indicate keratins genes on the same chromosome/genomic scaffold. Black filled figures: intact genes; empty figures: pseudogenes; grey filled figures: non-keratin flanking genes; KAPs: keratin-associated proteins; shaded rectangle: hair-type keratins. NG: New Genes. The bold lines above some genes indicate those evolving in a concerted manner. Brackets indicate a sister relationship among genes, when clear established in the phylogenetic tree. Hair-type keratins are indicated by blue color.

Hair is one of the defining features of mammals, where it plays a crucial role in heat retention, sexual dimorphism, attraction of mates, skin protection, and to sense the immediate surroundings [1]. It seems to have evolved after the divergence of the therapsid lineage (leading to mammals) from the sauropsid lineage (reptiles, birds) approximately 310 to 330 million years ago [2, 3]. The major components of hair are alpha-keratins and keratin associated-proteins (KAPs), each of which is encoded by separate gene families [4, 5]. The alpha-keratin gene family is nested within the type I (acidic) and type II (basic) gene clusters that are located in two different chromosomes, whereas the KAPs subfamilies are embedded in the type I keratin gene domain [6] (Figure 1). The type I and II keratin genes are expressed in a wide variety of epithelia from different tissues [7].

Among mammals, the number and nature of genes associated to the alpha-keratin gene family may vary. For example, in humans the type I keratin gene cluster possess 11 hair-type genes (K23, K39, K40, K33a, K33b, K34, K31, K37, K38, K32 and K35) and 17 other type I keratin genes (Figure 1), whereas in the type II gene cluster humans possess 6 hair-type (K81, K86, K83, K85, K84 and K82) and 21 other type II keratin genes (Figure 1). All hair-keratins are expressed in the hair shaft, and a subgroup of them is also expressed in claws and nails [4]. The critical role of hair-keratins in hair biology is revealed by the fact that a variety of epidermal diseases are caused by mutations in single hair-keratin genes [5]. Additionally, deletion or mutation of genes encoding basic epithelial keratins also may result in hair loss in mice [9].

Although the appearance of hair is one of the key evolutionary events in mammalian evolution, its origin and evolution remains speculative [10, 11]. According to [12], type I and type II keratin clusters evolved from two genes that were present in the last common ancestor of vertebrates. Subsequently, during the water-to-land transition in the stem lineage of tetrapods, a major expansion and functional diversification of keratin genes occurred. These duplications were crucial in acquiring new functions, in skin or appendages, in response to the new terrestrial lifestyle [12]. Within mammals, [13] compared the type I and II keratin gene clusters of the opossum and human and found that the keratin gene clusters of the opossum seem to lack pseudogenes, and display a slightly increased number of genes. Furthermore, the opossum keratin genes were longer in comparison to human and also show longer intergenic distances. In both clusters of both species, hair-keratin genes present an interior position and they propose that these genes arose by multiple duplication and sequence drift [13].

In mammals hair is found in all species at least at some point during their lives [10]. Of the ca. 5,000 extant mammalian species, only a few lack fur, and the absence of hair appears to be consistent with the animal’s natural environment, such as in the cetacean case. Although the appearance of hair in early mammalian evolution contributed to the ecological diversification of terrestrial species [14], the hair loss in cetaceans was also a key evolutionary innovation to live in the aquatic environment, since the lack of fur improves their hydrodynamic and subaquatic movements.

By taking advantage of available complete sequenced genomes from mammals, comparative analysis of genomic sequence from two cetacean species (bottlenose dolphin *Tursiops truncatus* and minke whale *Balaenoptera acustorostrata*) and terrestrial relatives should provide insight into the evolution of the alpha-keratin genes in mammals. More specifically, a comparison of the genomic structure of the cetacean alpha-keratin type I and type II gene clusters with that of other laurasiatherian mammals should allow inferences regarding the evolutionary processes that gave rise to the hairless phenotype in cetaceans. Accordingly, the main objective of this study is to characterize the gene repertoire of the type I and type II alpha-keratin gene clusters of laurasiatherian mammals, and to assess the evolutionary pattern of the alpha-keratins gene families, with the goal of revealing the underlying basis for mammalian hair phenotypic diversity, especially in the hairless cetacean lineage. Results from our analyses revealed a dynamic evolutionary history, with lineage specific gene duplication and loss, and an interesting pattern of increased pseudogenization of hair type keratins in the cetacean lineage when compared to terrestrial counterparts, which as suggested by the “less-is-more” hypothesis, could have been an evolutionary adaptation associated to the transition of a terrestrial to an aquatic environment in this lineage.

## Results and discussion

### Genomic structure of the laurasiatherian keratin gene family

In order to characterize the gene repertoire present in the keratin gene clusters in laurasiatherian mammals, we manually annotated keratin genes in the genomes of representative species of all major lineages for which genomic information was available. Additionally, the mouse and human clusters were also annotated as outgroup species.

In all species examined synteny is conserved, indicating that genomic regions containing these gene families have been conserved in boreoeutherian mammals. Type I keratins are flanked by *SMARCE1* and *EIF1* genes, whereas, type II keratins by *FAIM2* and *EIF4B* genes (Figure 1). The exceptions to this pattern are the 5’ flanking regions of the horse and microbat, where the *C12orf44* gene, instead of the *FAIM2*, is located upstream of the type II gene cluster. In the case of the horse the *FAIM2* gene is located much more upstream in the same chromosome, whereas in the bat is located in a different scaffold. In the case of the dolphin both flanking genes could not be identified, but it is probably due to the incompleteness of its genome assembly (Figure 1). The cluster sizes among the studied species remain similar for both clusters (data not shown).

Regardless the incompleteness of the dolphin and minke clusters, our results present a comprehensive screening for keratin genes of eight mammals and an improved picture on the mammalian genomic organization for this gene family (Figure 1). Among the fur mammals included, this gene family is rather conserved and we could identify most keratin members or at least parts of most keratin genes across their genomes, where evidence for gene duplication and gene loss events throughout pseudogenization are restricted to few genes. The number of functional type I keratin genes ranged from 25 in microbat to 29 in the dog, and from 27 in human, mouse, cow and horse to 28 in microbat and dog in cluster II (only complete clusters were considered). Previous studies already pointed out the striking similarity in keratin clusters organization among placentals, marsupials and monotremes [12, 13] suggesting that the last common ancestor of mammals should have had a diverse repertoire of keratin genes. According to these studies, ancestral mammals had a similar range and spectrum of hair characteristics as is seen in modern species, but these studies only included human and mouse as placental representatives. By including laurasiatherian representatives, our study yields more support to this view, and reveals a more detailed history of lineage-specific gene duplications and deletions. When we take into account only the keratin genes of the species included in this study (i.e. excluding the missing part of clusters that we could not characterize in the dolphin and minke), there is evidence for a keratin gene loss due to pseudogenization in the hairless cetacean lineage relative to the other terrestrial mammals, particularly among the hair-type keratin genes (Figure 1).

### Orthologous relationships

**Figure 2:**
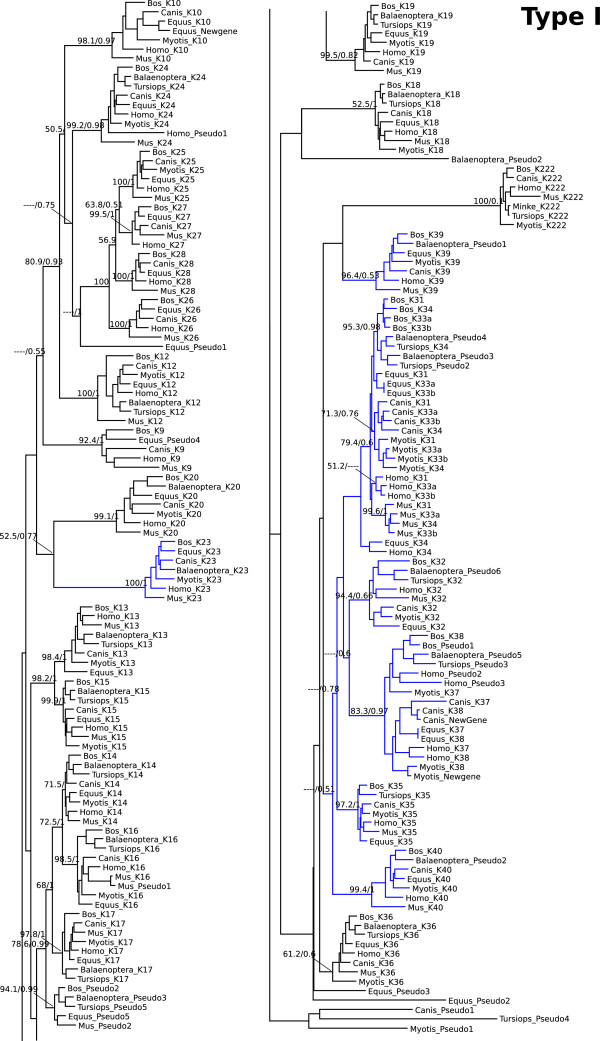
**Phylogenetic tree of type I keratins.** Maximum likelihood phylogram describing phylogenetic relationships among the type I keratin genes. Numbers above the nodes correspond to maximum likelihood bootstrap support values, and numbers below the nodes correspond to Bayesian posterior probabilities. Branches in blue indicated hair-type keratins.

**Figure 3:**
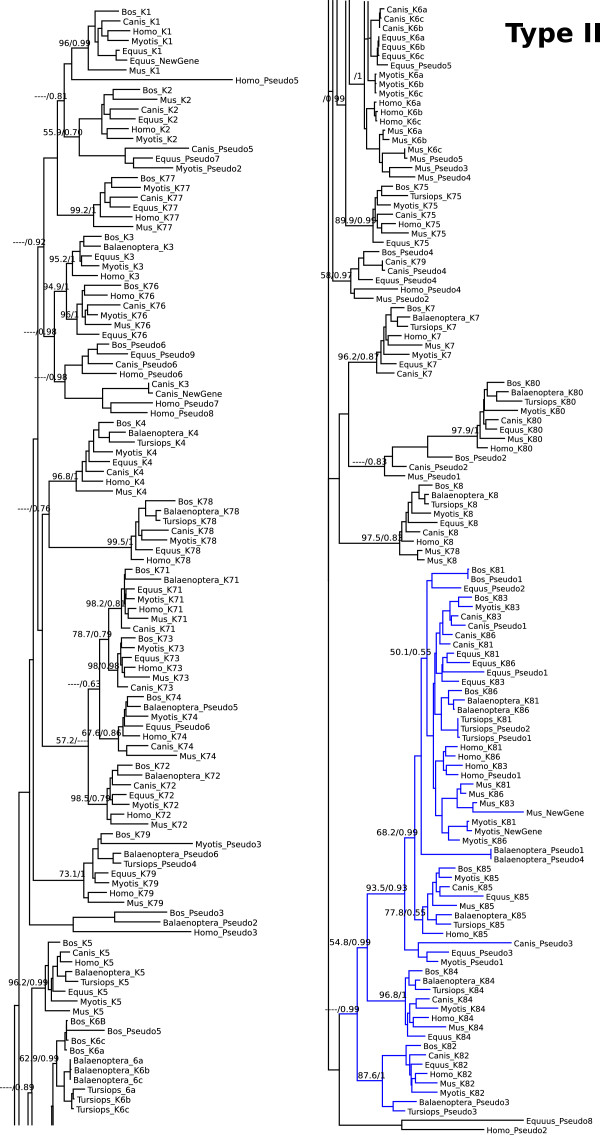
**Phylogenetic tree of type II keratins.** Maximum likelihood phylogram describing phylogenetic relationships among the type II keratin genes. Numbers above the nodes correspond to maximum likelihood bootstrap support values, and numbers below the nodes correspond to Bayesian posterior probabilities. Branches in blue indicated hair-type keratins.

Maximum likelihood and Bayesian phylogenies arranged type I and type II genes into well-supported clades (Figures 2 and 3), being possible to define the orthologous relationships. Given the small length of some pseudogenes (i.e. few exons identified), and their high evolutionary rate that may obscure their phylogenetic history, the identity of some pseudogenes were determined according to their location in the genome rather by their position in the phylogenetic tree.

In cluster II, whereas cow, dog, bat, horse, mouse and human present all hair-type keratin genes, two out of seven hair-type genes (K83 and K82) were exclusively lost in cetacean lineage, and one more (K86) exclusively in dolphin lineage (Figure 1). Interestingly, [15] reported that the loss of K81, K86 and K83 hair-type keratin genes were responsible for the hairless phenotype of the Hirosaki hairless rat. Regarding the other type II keratins, the cetacean species also exclusively lost K79, and the minke whale is the only species where K75 is a pseudogene (Figure 1). The K3 and K78 genes were lost in mouse lineage (Figure 1). Regarding the other species, the K74 is a pseudogene in horse and minke lineages (Figure 1). Additionally, we could identify one new intact keratin, located between K7 and K81 in mouse and microbat lineages, another new keratin located between K77 and K76 in dog lineage, another one between K1 and K77 in horse lineage. Also there is one new keratin gene between K79 and K8 in mouse lineage. This keratin gene is recovered within the K8 clade, and not with K78, indicating that this gene derived from K8 duplication, whereas the K78 was lost. The extra keratin gene identified between K7 and K81 in the mouse lineage was already described by [16], but there is no other report for the new type II keratins identified in microbat, dog and horse genomes, neither for the extra mouse keratin located between K79 and K8. The phylogeny indicates that all extra keratins aforementioned resulted from independent duplications in each lineage, and there is no corresponding ortholog in other species (Figure 3).

Regarding the hair-keratins in cluster I, K37 and K38 are completely absent from the mouse genome (Figure 1). In both minke and dolphin, K38 was completely lost whereas K31 and K37 are still present as pseudogenes. In cow, K37 keratin also is a pseudogene, suggesting that its functional lost occurred in the last common ancestor of artiodactyls. Besides these losses shared with the dolphin, the minke whale has lost almost all the hair-type keratins from type I cluster, only the K23 is still present. Whereas the dolphin still has K34, K32 and K35 genes, minke whale does not. Moreover, the minke whale completely lost the K33a and K33b genes, and the K39 and K40 are pseudogenes, but this genomic portion of cluster is missing in the dolphin genome, so we cannot know whether it is a minke specific loss or if it is shared by both cetacean species. In dog and microbat an extra hair-keratin gene located between K37 and K38 was found, and they were originated from independent duplications in each lineage (Figure 2). Regarding the other type keratins in cluster I, minke, dolphin, microbat and horse lost K9 gene. K26 is a pseudogene in microbat, and the horse is the only species where K222 is a pseudogene. Also there is a new keratin gene in the horse lineage, located between K28 and K10, with no corresponding ortholog in other species (Figure 1).

### Mode of gene family evolution

The evolutionary patterns observed in the keratin gene family can be attributed to a mixture of divergent, concerted, and birth-and-death evolutionary models [17]. The divergent model characterized most of the keratin genes analyzed in this study (Figures 2 and 3), this can be seen in our phylogenetic analyses as orthologous genes are recovered in clades in the species where the genes were identified. The concerted evolution, i.e. members of a gene family evolving in a concerted manner rather than independently, can be observed to occur in Type I keratin cluster, among the K33a, K33b, K34 and K31 genes (Figure 2), and in Type II cluster among the K6a, K6b and K6c and K81, K83 and K86 genes (Figure 3). This can be seen in Figures 2 and 3, where the phylogenetic trees recover paralogous instead of orthologous clades, indicating that paralogous genes are more similar to one another than they are to their true orthologous in closely related species. This pattern is typically attributed to the homogenizing effects of interparalog gene conversion or unequal crossing-over [18].

Also, our analysis of the keratin gene families reveals characteristics consistent with the birth-and-death mode of evolution: we documented lineage-specific patterns of duplication and differential deletion and retention of genes among lineages. Consequently, some species possess keratin genes that have no orthologous counterparts in closely related species whereas in some cases, the ortholog of an apparently functional gene in one species is a pseudogene in another species (Figure 1).

It has been suggested that changes in regulatory sequences rather than changes in protein-coding regions would be mainly responsible for the phenotypic differences among species [19]. Nevertheless, the differential loss and retention of genes among different mammalian lineages may also constitute an important source of adaptive change through the “birth” of new genes via duplication and the “death” via inactivation or deletion. Changes in gene family size have been important during mammalian evolution [20–24], and large differences in gene family size are generally attributed to a selective advantage for either an increased or decreased gene number [25–28].

Results of our study reveal a higher rate of keratin gene loss due to primarily pseudogenization and secondarily to deletion in the cetacean lineage, especially of the hair-type keratin genes, when compared to the fur terrestrial relatives. It is likely that changes in keratin gene numbers in mammals could explain differences in the observed hair morphology among species, as is the case that the hairless species presents an extensive gene loss. The idea that gene loss can be a mechanism for adaptive evolutionary change was first introduced by Olson [29]. The “less-is-more” hypothesis suggests that gene loss can be adaptive in some cases, although it is intuitive to consider gene loss as neutral or non-adaptive, and to associate adaptation with the gain of new genes [29]. This can be particularly feasible in certain scenarios such as a changing environment, as the cetacean case, where a loss of function can also bring a selective advantage because having no hair when living exclusively in an aquatic environment would improve their subaquatic movements.

In a recent paper in which the minke whale genome was analyzed, the authors briefly reported that, compared to other mammalian species, the minke whale and the bottlenose dolphin had evolutionarily contracted keratin-related genes (among KRT and KAPs gene families) [30]. However, they didn’t specifically discriminate the hair-type genes from the other keratin genes, neither presented which genes would have been lost.

The “less-is-more” hypothesis has been unappreciated and little explored, but there is evidence that strongly support this view. Most well known examples are found in Bacteria and Archaea [31, 32], but there are also examples of adaptive gene losses in vertebrates, such as the loss of different opsin genes in different vertebrate lineages as a response to selective pressures imposed by new specific habitats [33].

Gene loss can arise in response to change in the pattern of selective pressures, and that is probably what happened in early evolution of cetaceans regarding the keratin gene family. Although adaptive gene loss cannot be evaluated directly, comparative approaches have been useful in linking events of gene losses and the loss of traits. Another good example of increased level of pseudogenization in the cetacean lineage was shown for the olfactory receptor gene family [34]. Evolution of the olfactory receptors genes in toothed whales features a multitude of independent pseudogenization events, supporting anatomical evidence that these animals have lost their olfactory sense.

The precise molecular mechanisms underlying hair loss are still unclear, but great advances have been done. A recent study [35] investigated the roles of the *hairless* (*Hr*) gene and the *fibroblast growth factor 5* (*FGF5*), to determine whether evolutionary changes in these two genes were associated with the hair loss of cetaceans during the transition from land to water. Their results suggest that the cetacean *Hr* gene has experienced a significant relaxation of functional constraints during its evolution from terrestrial mammals and subsequent diversification, and probably lost its function, whereas the *FGF5* was under a positive selection regime. The results from [35] together with the results of the present study shed light on the molecular basis of hair loss, and indicate that these molecular mechanisms are more complex and do not rely on the evolutionary change of only one gene. Indeed, additional genes related to hair development should be investigated to improve our understanding of the hair loss phenotype in mammals, and also further taxa should be added to the discussion (such as other fully aquatic marine mammals, as sirenians).

To further test whether the hair-type keratin genes in cetaceans which are still functional show a high degree of divergence, we performed relative rate tests [36] in which we compared the rates of evolution of the cetacean sequence and its closest relative in our study (cow), using the human sequence as outgroup. These analyses were performed in Mega6 [37]. Briefly, the results showed that for three hair-type keratin genes (K23, K32 and K84) the rate of evolution between the cetacean species and the cow was not significantly different (P = 0.35, 1.00 and 0.44, respectively). For the other four hair-type keratin genes (K35, K81, K85 and K86), we found differences in the rate of evolution, suggesting that these cetacean hair-type keratin genes may be under reduced selective constraints (P < 0.01, for all genes).

## Conclusion

This study provides a comprehensive characterization of alpha-keratin genes among laurasiatherian mammals and shed light on the mechanisms involved in the evolution of this gene family. Differences in gene family size are due to lineage-specific gene duplication and gene loss may provide clues to the evolutionary forces that have shaped mammalian genomes and also provide a better understanding of some of the major evolutionary transition such as the return to the sea undergone by cetaceans. Our results show increased rates of gene loss in the cetacean lineage when compared to terrestrial counterparts and can provide comparative evidence the “less-is-more” hypothesis. Interestingly, some of the gene losses we observed are shared by both species examined in this study, suggesting that they are the most likely candidates to explain the hairless phenotype in this group of mammals. Future functional studies should be performed using these genes to test this prediction.

By the time that this paper was being reviewed a very interesting paper was accepted to be published in this journal. This paper characterized the keratin associated proteins (KAPs or KRTAPs) gene family in several mammalian genomes, and notably, also reported a highest pseudogenization rate in the hairless dolphin [38]. The authors speculate that the large number of KAP pseudogenes observed in dolphin relative to intact genes in other terrestrial mammals, was likely to have occurred in response to adaptation to ecological pressures. Also they suggest that changes in KAPs can be related to morphological diversity on hair phenotypes. Taken both studies together, we can conclude that both alpha-keratins (KRT) and keratin associated proteins (KAPs or KRTAPs) play an important role in diversification and evolution of hair phenotype in mammals.

## Methods

### DNA Sequence data

The human keratins were retrieved from the Human Intermediate Filament Database (http://www.interfil.org). The unnanotated genomic sequences of the keratin gene clusters from dolphin (*Tursiops truncatus*), cow (*Bos taurus*), dog (*Canis familiaris*), horse (*Equus caballus*), little brown bat (*Myotis lucifugus*) and mouse (*Mus musculus*) were obtained in Ensembl database. Keratin gene sequences were identified and annotated by comparing known human sequences to genomic contigs using the program BLAST2 [39]. Human keratins were named according to the revised keratin nomenclature [38]. An open intact reading frame with the canonical structure typical of vertebrate keratin characterized putatively functional genes, whereas pseudogenes were identifiable because of their high sequence similarity to functional orthologs and the presence of inactivating mutations, and/or the lack of exons. Accession numbers are listed in Additional file 1. Phylogenetic relationships among Laurasiatherian mammals for comparative inference were derived from [40].

### Phylogenetic inference

Phylogenetic relationships among the different members of the keratin gene family in the dataset were estimated using Bayesian and maximum likelihood approaches, as implemented in Mr. Bayes v3.1.2 [41] and Treefinder version March 2011 [42], respectively. Type I and type II keratin clusters were analyzed separately. The nucleotide sequences were aligned using the program MUSCLE [43]. The best fitting model of nucleotide substitution was estimated using the “propose model” routine from Treefinder version March 2011. For the Bayesian analyses, two simultaneous independent runs were performed for 30,000,000 iterations of a Markov Chain Monte Carlo algorithm, with six simultaneous chains, sampling every 1,000 generations. Support for the nodes and parameter estimated were derived from a majority rule consensus of the last 15,000 trees sampled after convergence. In maximum likelihood, we estimated the best tree for each cluster, and support for the nodes were obtained with 1,000 bootstrap pseudoreplicates.

### Availability of supporting data

The data sets supporting the results of this article are available in the TreeBase repository, http://purl.org/phylo/treebase/phylows/study/TB2:%20S16426.

## Electronic supplementary material

Additional file 1: **Accession numbers and sources of the retrieved keratin sequences for the species included in this study.** (DOC 88 KB)

## Abbreviations

KAPs: Keratin associated-proteins.
