# Supplementary material for: Increased rate of hair keratin gene loss in the cetacean lineage
Source: BMC Genomics. 2014 Oct 6;15(1):869. doi: 10.1186/1471-2164-15-869 (PMC4195889; doi:10.1186/1471-2164-15-869)
Supplement: Supplementary file 1 — Additional file 1: Accession numbers and sources of the retrieved keratin sequences for the species included in this study. (DOC 88 KB) [file 12864_2014_6533_MOESM1_ESM.doc]

Additional file 1. Accession numbers and sources of the retrieved keratin sequences for the species included in this study.

|  |  | Type I | | | Type II | | |
| --- | --- | --- | --- | --- | --- | --- | --- |
| Common name | Species | Keratin | Accession number | Source | Keratin | Accession number | Source |
| Human | *Homo* | K9 | NM_000226.3 | GenBank | K1 | NM_006121.3 | GenBank |
|  | *sapiens* | K10 | NM_000421.3 | GenBank | K2 | NM_000423.2 | GenBank |
|  |  | K12 | NM_000223.3 | GenBank | K3 | NM_057088.2 | GenBank |
|  |  | K13 | NM_153490.2 | GenBank | K4 | NM_002272.2 | GenBank |
|  |  | K14 | NM_000526.4 | GenBank | K5 | NM_000424.3 | GenBank |
|  |  | K15 | NM_002275.3 | GenBank | K6a | NM_005554.3 | GenBank |
|  |  | K16 | NM_005557.3 | GenBank | K6b | NM_005555.3 | GenBank |
|  |  | K17 | NM_000422.2 | GenBank | K6c | NM_173086.4 | GenBank |
|  |  | K18 | NM_000224.2 | GenBank | K7 | NM_005556.3 | GenBank |
|  |  | K19 | NM_002276.4 | GenBank | K8 | NM_002273.3 | GenBank |
|  |  | K20 | NM_019010.2 | GenBank | K71 | NM_033448.2 | GenBank |
|  |  | K23 | NM_015515.3 | GenBank | K72 | NM_080747.2 | GenBank |
|  |  | K24 | NM_019016.2 | GenBank | K73 | NM_175068.2 | GenBank |
|  |  | K25 | NM_181534.3 | GenBank | K74 | NM_175053.3 | GenBank |
|  |  | K26 | NM_181539.4 | GenBank | K75 | NM_004693.2 | GenBank |
|  |  | K27 | NM_181537.3 | GenBank | K76 | NM_015848.4 | GenBank |
|  |  | K28 | NM_181535.3 | GenBank | K77 | NM_175078.2 | GenBank |
|  |  | K31 | NM_002277.2 | GenBank | K78 | NM_173352.2 | GenBank |
|  |  | K32 | NM_002278.3 | GenBank | K79 | NM_175834.2 | GenBank |
|  |  | K33a | NM_004138.2 | GenBank | K80 | AJ717743.1 | GenBank |
|  |  | K33b | NM_002279.3 | GenBank | K81 | NM_002281.3 | GenBank |
|  |  | K34 | NM_021013.3 | GenBank | K82 | NM_033033.3 | GenBank |
|  |  | K35 | NM_002280.4 | GenBank | K83 | NM_002282.3 | GenBank |
|  |  | K36 | NM_003771.4 | GenBank | K84 | NM_033045.3 | GenBank |
|  |  | K37 | NM_003770.4 | GenBank | K85 | NM_002283.3 | GenBank |
|  |  | K38 | NM_006771.3 | GenBank | K86 | NM_002284.3 | GenBank |
|  |  | K39 | NM_213656.3 | GenBank |  |  |  |
|  |  | K40 | AJ786658.1 | GenBank |  |  |  |
| Bottlenose | *Tursiops* |  | Scaffold 115381 | Ensembl |  | Scaffold 1393 | Ensembl |
| dolphin | *truncatus* |  | Scaffold 105773 | Ensembl |  | Scaffold 2647 | Ensembl |
|  |  |  | Scaffold 24 | Ensembl |  |  |  |
|  |  |  | Scaffold 85365 | Ensembl |  |  |  |
|  |  |  | Scaffold 105081 | Ensembl |  |  |  |
| Minke whale | *Balaenoptera acutorostrata* |  | NW_006726421.1 | GenBank |  | NW_006726687 | GenBank |
| Microbat | *Myotis* |  | GL429913 | Ensembl |  | GL429858 | Ensembl |
|  | *lucifugus* |  | GL430096 | Ensembl |  |  |  |
| Cow | *Bos* |  | Chr. 19 | Ensembl |  | Chr. 5 | Ensembl |
| Dog | *Canis* |  | Chr. 9 | Ensembl |  | Chr. 27 | Ensembl |
|  | *familiaris* |  |  |  |  |  |  |
| Horse | *Equus* |  | Chr. 11 | Ensembl |  | Chr. 6 | Ensembl |
|  | *caballus* |  |  |  |  |  |  |
| Mouse | *Mus* |  | Chromosome 11 | Ensembl |  | Chr. 15 | Ensembl |
|  | *musculus* |  |  |  |  |  |  |
